# A prospective analysis of the long-term impact of the COVID-19 pandemic on well-being and health care among children with a chronic condition and their families: a study protocol of the KICK-COVID study

**DOI:** 10.1186/s12887-023-03912-7

**Published:** 2023-03-22

**Authors:** Petra Warschburger, Clemens Kamrath, Stefanie Lanzinger, Claudia Sengler, Susanna Wiegand, Julia M. Göldel, Susann Weihrauch-Blüher, Reinhard W. Holl, Kirsten Minden

**Affiliations:** 1 Department of Psychology, Counseling Psychology, University of Potsdam, Karl-Liebknecht-Str. 24-25, 14476 Potsdam, Germany; 2 Center of Child and Adolescent Medicine, University of Giessen, Gießen, Germany; 3 Institute of Epidemiology and Medical Biometry, ZIBMT, Ulm University, Ulm, Germany; 4 German Center for Diabetes Research (DZD), Munich-Neuherberg, Germany; 5 Deutsches Rheuma-Forschungszentrum (DRFZ), Program Area Epidemiology, Berlin, Germany; 6 Center for Social-Pediatric Care, Department of Pediatric Endocrinology and Diabetology, Charité Universitätsmedizin Berlin, corporate member of Freie Universität Berlin und Humboldt- Universität zu Berlin, Berlin, Germany; 7 Department of Pediatrics I, Pediatric Endocrinology, University Hospital Halle/S, Halle/S, Germany; 8 Department of Pediatric Respiratory Medicine, Charité-Universitätsmedizin Berlin, corporate member of Freie Universität Berlin und Humboldt- Universität zu Berlin, Immunology and Critical Care Medicine at Charité University Hospital Berlin, Berlin, Germany

**Keywords:** Chronic conditions, COVID-19, Children and adolescents, Parents, Risk perception, Psychosocial strain, Diabetes, Rheumatic diseases, Obesity

## Abstract

**Background:**

There is consistent evidence that the COVID-19 pandemic is associated with an increased psychosocial burden on children and adolescents and their parents. Relatively little is known about its particular impact on high-risk groups with chronic physical health conditions (CCs). Therefore, the primary aim of the study is to analyze the multiple impacts on health care and psychosocial well-being on these children and adolescents and their parents.

**Methods:**

We will implement a two-stage approach. In the first step, parents and their underage children from three German patient registries for diabetes, obesity, and rheumatic diseases, are invited to fill out short questionnaires including questions about corona-specific stressors, the health care situation, and psychosocial well-being. In the next step, a more comprehensive, in-depth online survey is carried out in a smaller subsample.

**Discussion:**

The study will provide insights into the multiple longer-term stressors during the COVID-19 pandemic in families with a child with a CC. The simultaneous consideration of medical and psycho-social endpoints will help to gain a deeper understanding of the complex interactions affecting family functioning, psychological well-being, and health care delivery.

**Trial registration:**

German Clinical Trials Register (DRKS), no. DRKS00027974. Registered on 27th of January 2022.

## Background

In Germany, around 10% of children and adolescents are affected by a chronic physical health condition, such as obesity, diabetes, or rheumatic disease [1–3]. Although children and adolescents seem to be less often and less severely affected by the coronavirus disease 2019 (COVID-19) compared to adults [4–9], children and adolescents with a chronic health condition (CC), such as diabetes, obesity, or inflammatory rheumatic disease, are assumed to be at higher risk of a more severe course of an infection with severe acute respiratory syndrome coronavirus 2 (SARS-CoV-2) [6, 10, 11]. Type 1 diabetes, for instance, ranks among the strongest risk factors for severe illness and hospitalization when infected with the SARS-CoV-2 virus [10], depending on the metabolic control [12], which often deteriorates in adolescence [13, 14]. Also, obesity belongs to the strongest risk factors for hospitalization with the SARS-CoV-2 virus. Various influence factors constitute this risk, most of all chronic inflammation, impaired immune response, and underlying cardiopulmonary disease [15]. Evidence regarding rheumatic diseases or a consequential therapeutically induced immunosuppression as a risk factor for severe infection with the SARS-CoV-2 virus show conflicting results [16–19]. At the same time, altered daily structure and, therefore, altered health behavior can impair the underlying rheumatic disease or obesity [15, 20]. In addition, these changes result in a higher prevalence of several chronic conditions, such as obesity [21]. Also, for diabetes, an increased prevalence has been observed during the COVID-19 pandemic [22].

Also in times without a pandemic, children and youth with CCs as well as their parents are at increased risk of developing mental health problems, such as anxiety, depression, and impaired health-related quality of life [23–29]. During the COVID-19 pandemic, children with CCs and their families are confronted with unique challenges in their disease management routines. The federal states in Germany have implemented swift, wide-ranging public health emergency measures that have included a national lockdown with social restrictions (e.g., stay-at-home orders) and quarantines to reduce interpersonal contacts. On March 13, 2020, all federal states in Germany closed kindergartens and schools; nearly all colleges and universities followed. School closures substantially disrupt the lives of children and their families and may have consequences for child health [30–34] and parental well-being [35–39]. In addition, Kindergartens and schools provide an essential source of meals and nutrition, health care, including behavioural health supports, physical activity, social interaction, support for students with special education needs and disabilities, and other vital resources for healthy development [40]. It is important to note that childhood and adolescence are periods of life characterized by heightened sensitivity to social stimuli and the increased need for peer interaction. The physical distancing measures have radically reduced opportunities to engage in social contacts outside of the household. Consequently, social deprivation during this sensible developmental period might have caused far-reaching consequences [41]. In addition, parents may have faced economic insecurity, had to educate their children at home as a substitute for school attendance and had to deal with an uncertain outlook into the future. Working at home and living with preschool-aged children has particularly influenced the extent of mental distress during the pandemic [42, 43]. Therefore, the impact of lockdowns implemented in response to COVID-19 on mental health has raised concerns [44, 45]. There is increasing empirical evidence underpinning the negative effects of the pandemic and the associated containment measures on the psychosocial well-being of children [33, 34, 46] and their parents [38, 39] in the general population. However, so far only few studies focused on the psychosocial situation of children and adolescents with CCs [47, 48]. Given that preexisting mental and physical health problems are associated with higher levels of anxiety and depression during the COVID-19 pandemic [49], it can be assumed that the COVID-19 pandemic represents an additional risk that will be more pronounced among those children and adolescents and their families who are facing preexisting physical, mental or social vulnerabilities [36, 50].

With respect to regular medical care, which is essential for children and adolescents with CCs, there were major changes. During the lockdown, in most medical institutions across the country, routine consultations took place alternatively by telephone or video contact. Socially and educationally disadvantaged populations might face more problems using telehealth services [36]. It is well known that threat appraisals (rating of the subjective vulnerability and severity of a disease risk) affect the adoption of health-protective behaviors and health care use [51]. In childhood, research has shown that in addition to the risk perceptions of the children themselves, parental threat appraisals also influence their intention to engage in preventive or intervention efforts [52, 53]. Reduced access to health care can be detrimental to pediatric health, and children with special needs are potentially at higher risk of severe illness due to a lack of health care than their healthy peers.

## Methods

### Objectives

**Fig. 1:**
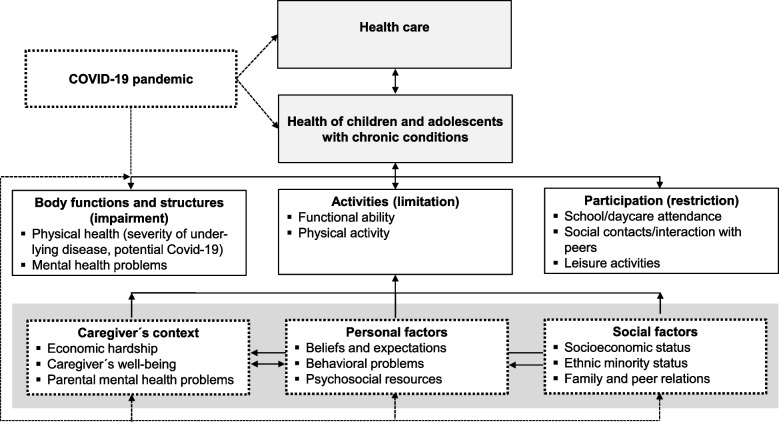
Conceptual model of the proposal considering the ICF structure (modified according to Cieza and Stucki [54])

Based on a comprehensive understanding of the complex interplay between medical condition, health care use, and environmental and specific context factors in CC (see Fig. 1 [54];), the overarching aim of the so-called KICK-COVID study is to examine the longer-term effects of the COVID-19 pandemic on both medical as well as psychosocial outcomes in children and adolescents with a CC and their families.

Based on the biopsychosocial model, the specific study objectives are to examine

1. the impact of the COVID-19 pandemic on the care of children and adolescents with a CC by analyzing deficiencies, interruptions and unmet needs in care and treatment,
2. the impact of the COVID-19 pandemic on body function and clinical outcomes (e.g., disease specific medical endpoints, physical and mental health), activities and participation of children and adolescents with different chronic diseases (diabetes, obesity, and rheumatic disease),
3. the psychosocial resources and risk perceptions of children and their parents facing the Corona pandemic and their influence on child’s psychosocial adjustment, mental and physical health,
4. the interplay of the physical and mental health of the children,
5. the course of physical and mental health over time,
6. the impact of a potential COVID-19 infection on these variables, and
7. disease-specific differences and similarities with respect to the impact of the COVID-19 pandemic.

### Study design

**Fig. 2:**
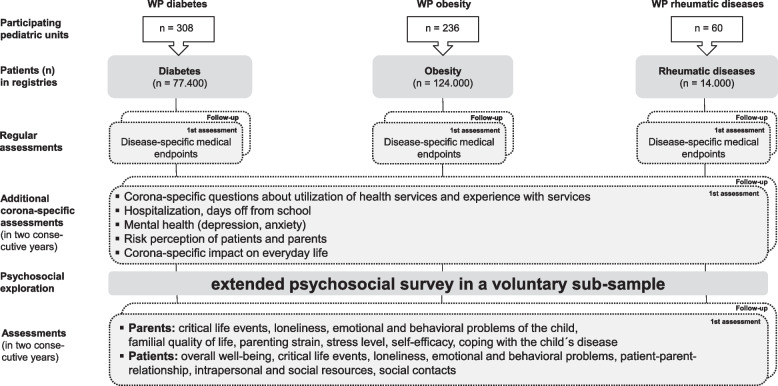
Illustration of the study design and recruitment process

The ongoing prospective observational study started recruitment in June 2021. A two-step approach (see Fig. 2) has been implemented: In the first step, additional COVID-19-specific questions have been added to the already existing surveys for the regular check-ups. Parents and adolescents provide data on their own psychosocial situation. In addition, parents assess as proxies the situation of their children under 12 years. Since this assessment is part of a comprehensive medical examination, only short economic instruments can be used in order to prevent exhaustive strain on the families. To get a deeper insight into the relevant psychological processes, a smaller voluntary subsample is asked to fill-in an extended online survey and forward it to their children (> 9 years) as well. After one-year, follow-up assessments will take place.

### Study participants

Parents and their underage children (aged up to 18 years) who are already taking part in any of the three patient registries for diabetes (German Diabetes Prospective Follow-up Registry, DPV [55]), obesity (German Obesity Prospective Follow-up Registry, APV [56]), or rheumatic disease (National Pediatric Rheumatology Database, NPRD [57]) are eligible for inclusion.

#### Recruitment

**Fig. 3:**
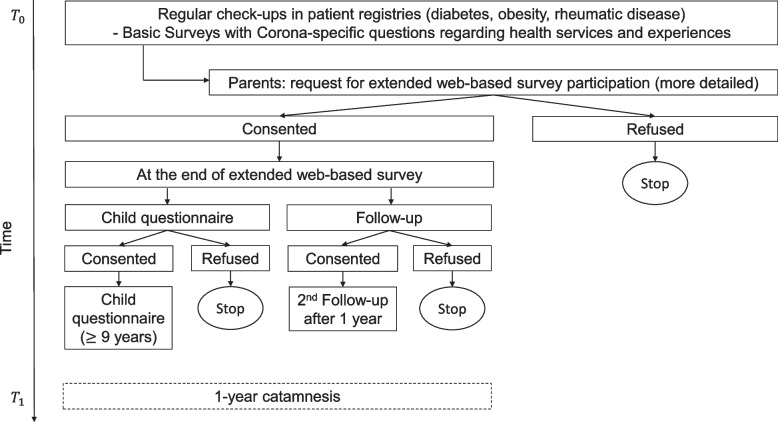
Recruitment flow chart

Recruitment takes place during the regular check-ups within the participating clinical facilities. Clinicians ask their adolescent patients and their accompanying parents to fill in a two-page short questionnaire during their waiting time; for children younger than 12 years, the questionnaire is only completed by the accompanying parent. After the completed questionnaires are handed over to the treatment team in the healthcare facility, the next step is to ask whether the relatives would also be willing to take part in a further, more extensive survey. If they agree, the families receive a flyer with information and a barcode that leads directly to the online questionnaire. At the end of the questionnaire, consent is obtained from the parents to be contacted again after 1 year. In addition, the parents are asked for parental consent for the participation of the underage child (from about 9 years of age). If the parents agree, the children also receive an online questionnaire at the e-mail address provided and can then fill out the children’s questionnaire themselves. At the end of the respective questionnaires, parents and their children can download quizzes, an audio-guided relaxation training [58], small puzzles and colouring books, or take part in a computer game on risk-taking behavior as an incentive. In order to recruit a high number of eligible participants, it is planned to realize the whole recruitment process over a period of 1 year. The whole recruitment process is depicted in Fig. 3.

### Study measures

This survey uses several questionnaires and self-constructed items to fulfil the primary study objectives stated above.

#### Basic survey

An overview of the study measures applied in the basic surveys is given in Table 1.

**Table 1:** Overview of corona-specific study measures in the basic survey

Study Measures: Basic Questionnaires	Parents^**a**^	Parents^**b**^	Teenagers
**Emotions and psychological distress during the COVID-19 pandemic**			
COVID-19 pandemic-specific impact on parental well-being and everyday life^c^	x	x	
COVID-19 pandemic-specific impact on well-being and everyday life of the afflicted child^c^	x^d^		x
**Psychosocial adjustment**			
Health-related strain^c^	x	x	x
Perceived stress of the afflicted child^c^	x		x
Perceived loneliness of the afflicted child^c^	x		x
Media usage			x
Well-Being Index WHO-5	x^d^		x
General Anxiety Disorder-Scale GAD-7			x
Patient Health Questionnaire PHQ-9			x
School experience^c^			x
**Disease-specific healthcare use**			
Corona-specific utilization of health services in the last 12 months^c^	x	x	

^a^Parents of afflicted children younger than 12 years

^b^Parents of afflicted children aged 12 years and older

^c^self-constructed questionnaire

^d^proxy-report

#### Basic survey: Common measures for parents and children

Several measures are administered for the data collection. While parents are asked to fill out the questionnaire for their children under the age of 12, they answer an additional separate questionnaire when their afflicted child is older than 12. The following assessments are identical for the parents of these two age groups.

##### COVID-19 pandemic-specific impact on well-being and everyday life

Based on experience from a previous study [59], a self-constructed 6-item scale is administered to assess how the COVID-19 pandemic has impacted the well-being of afflicted children and their families in different aspects of their every-day life. Parents are asked to rate the quality of several life domains in light of the COVID-19 pandemic: the care for the child at home, the treatment of the child’s condition, the occupation, the financial situation, and the partnership; with scales ranging from 0 “totally bad” to 10 “totally good”. Furthermore, participants are asked to indicate how stressed they feel due to the COVID-19 pandemic with a scale ranging from 0 “not stressed at all” to 10 “totally stressed”.

##### Health-related strain

Two self-constructed items [59] are used to assess health-related strain in the light of the COVID-19 pandemic. On two 11-point Likert scales, participants are asked to (i) state how dangerous they consider a COVID-19 infection in their afflicted child, ranging from 0 “totally harmless” to 10 “totally dangerous”, and (ii) rate the intensity of their fear for the child’s health, with 0 indicating “no fear at all” and 10 indicating a very intense fear.

##### Disease-specific health care use

A total of 10 self-constructed items are implemented to assess the corona-specific utilization of health services in the past 12 months. Parents are asked to provide information about the frequency of disease-specific health care visits; medical appointment cancellations and their causes; and the provision and quality of alternative virtual office hours.

#### Basic survey: Common measures for children and adolescents

For the questionnaires aiming at the data collection in children under the age of 12 and adolescents aged 12 years or older, the following measures are applied.

##### Perceived stress

A self-constructed item aims to assess how stressed the child feels because of the COVID-19 pandemic with an 11-point numerical rating scale ranging from 0 “not stressed at all” to 10 “totally stressed”. As described above, for children under the age of 12, the accompanying parent is asked to rate this item for their child.

##### Perceived loneliness

To measure how lonely a child feels due to the COVID-19 pandemic, a self-constructed item on a Likert scale ranging from 0 to 10 is applied. Older children are asked to select a response ranging from “not stressed at all” to “totally stressed”, and the parents answer this item for younger children.

##### COVID-19-specific well-being in everyday life

A self-constructed 5-item scale based on Warschburger et al. [59] aims to assess the child’s well-being across several life domains. Children from 12 years are asked to rate the current state of their: family, education/vocational education/occupation, friends, leisure time, and illness. The scales range from 0 “totally bad” to 10 “totally good”. These items are posed to the parents of children when they are younger than 12 years. The German version of the World Health Organization Five Well-Being Index (WHO-5) is additionally administered to measure the current general well-being (e.g., “I was happy and in a good mood”) of children and adolescents during the past 2 weeks [60, 61]. The WHO-5 has a 5-item Likert scale ranging from 0 “at no time” to 5 “all of the time” and has an internal consistency of Cronbach’s α = .89 to Cronbach’s α = .92 [60, 62].

#### Basic survey: Measures for adolescents

##### School experience

Two items have been constructed for the purpose of assessing the educational experiences of adolescents during the COVID-19 pandemic. One multiple-choice question aims to assess the teaching method that the students encountered during the last 2 weeks, with possible answers comprising home-schooling, classroom teaching and frequency, holidays, or having finished school. The other item asks, if applicable, how students have succeeded in learning at home, with a Likert scale ranging from 0 “totally badly” to 10 “totally well”.

##### Perceived risk

The risk perception of adolescents is measured with three self-constructed items [39]. Adolescents are asked to state how dangerous they consider a COVID-19 infection to be for (i) themselves, and (ii) others on an 11-point Likert scale. Furthermore, adolescents indicate (iii) their perceived risk of a coronavirus infection, on a scale ranging from 0 (“totally unlikely”) to 10 (“very likely”).

##### Media usage

A 5-point Likert scale established by another survey [63, 64] is administered to assess the electronic media usage of teenagers. The adolescents are asked to report how much time they spend on average during the day with watching TV/videos, a gaming console, using the computer/internet, listening to music, their phone; answers range from “not at all” to “more than 4 hours”.

##### Anxiety

Anxiety symptoms (e.g., not being able to stop or control worrying) during the past 2 weeks are measured with the German version of the General Anxiety Disorder-Scale (GAD-7) [65, 66]. The 4-point Likert scale consisting of seven items ranges from 0 “not at all” to 3 “nearly every day”. The GAD-7 has an internal consistency of Cronbach’s α = .79 to Cronbach’s α = .91 [67] and studies reported a successful usage in adolescents [68, 69].

##### Depression

To detect depressive symptoms during the past 2 weeks (e.g., feeling down, depressed, or hopeless), the German version of the 9-item Patient Health Questionnaire (PHQ-9) [70] is administered as a screening inventory. The 4-point Likert scale of the PHQ-9 ranges from 0 “not at all” to 3 “nearly every day” and has an internal consistency of Cronbach’s α = .88 [71].

#### Web-based extended psychosocial survey

In addition to the baseline survey, parents and with their consent also the children (aged > 9 yrs.) are invited to fill a more comprehensive psychosocial web-based survey. Table 2 summarizes the questionnaires used in the parental and child assessment.

**Table 2:** Overview of study measures in the web-based survey

Study Measures: Web-based, extended Questionnaires	Parents	Teenagers
**Socio-demographic and medical assessment**		
General socio-demographic factors^b^	x	x
Factors related to the chronic condition^b^	x	x
MacArthur Scale	x	
Winkler-Index^a^	x	
**Emotions and psychological distress during the COVID-19 pandemic**		
COVID-19 pandemic-specific impact on parental well-being and everyday life^b^	x	x
Corona-specific burden^a^	x	x
**Psychosocial adjustment**		
Self-Assessment Manikin SAM	x	x
Well-Being Index WHO-5	x	
Patient Health Questionnaire PHQ 4	x	
Perceived-Stress Scale PSS-4	x	
De-Jong-Gierveld-Skala	x	x
Strengths and Difficulties Questionnaire SDQ	x^a^	x^a,c^
Child Health Questionnaire CHQ	x	
KIDSCREEN-27^a^		x
**Coping and resources**		
Coping orientation to problems experienced Brief COPE	x	
General self-efficacy scale (ASKU)	x	
Scale for the assessment of internal and external sense of control (IE-4)	x	
OSLO social support scale OSLO	x	
Child’s coping	x	
Child’s ressources^b^	x	
Coping with a disease questionnaire CODI		x
Questionnaire of Resources in Childhood and Youth FRKJ-8-16^a^		x
Social integration^a^		x
Corona-specific self-efficacy		x
**Family relations and interactions**		
Potentially harmful parenting behavior	x	
Family-related life questionnaire FLQ^a^	x	
Brief Parental Burnout scale BPBS	x	
Parental-Representation-Screening-Questionnaire EBF-KJ^a^		x
Corona-specific parental support^b^		x
**Control Variables**		
Social desirability scale (SEA-K)	x	

^a^modified

^b^self-constructed

^c^child-version

#### Web-based common measures for parents and children

##### Sociodemographic and medical assessment

Two self-constructed items are implemented asking the participants about their age and gender. The child questionnaire includes a third item assessing the adolescents’ living situation (“Where do you currently live?”), while the parental questionnaire incorporates three additional questions regarding the child’s year of birth, weight and height, and location of their child’s treatment.

Furthermore, items adapted from Warschburger [59] measure CC-specific medical variables. On a 5-point scale, participants are asked to rate the subjective severity, long-term effect of the CC, and impact of the COVID-19 pandemic on the child’s CC. A fourth item, included in the child questionnaire assesses how well the child is coping with the CC in general, with a scale from 0 = “not at all “to 4 = “very well“.

##### COVID-19-specific questions

COVID-19-specific questions are included to assess the COVID-19 related impact and burden experienced by the participants. Two self-constructed items adapted from Warschburger et al. [59] ask the children and parents to indicate whether they have faced a COVID-19 infection and rate its severity on a 4-point scale ranging from “symptom-free” to “severe”. The parental survey includes 30 additional self-constructed items, assessing, e.g., if their afflicted child suffered from the long-COVID syndrome, or whether they and their child received COVID-19 vaccinations.

Modified items based on Calvano et al. [39] further measure the subjective burden on several areas of everyday life caused by the COVID-19 pandemic, such as the children’s education or the parents’ work. On a 5-point Likert scale, participants are asked to rate how burdensome they experienced COVID-19 with higher ratings indicating a higher burden. In addition, participants are asked to assess what has caused the most stress and anxiety and what has changed positively during the pandemic.

##### Psychosocial adjustment: Overall well-being

Two picture-based items using the Self-Assessment Manikin [72] are implemented assessing how the parents and children feel in general, and how stressed they are.

##### Psychosocial adjustment: Loneliness

The 6-item short version of the De-Jong-Gierveld-Skala [73] is administered to measure the children’s and parents’ overall loneliness. Statements relating to social and emotional loneliness are evaluated on a 4-point scale, with higher scores indicating more frequent loneliness. The questionnaire has an internal consistency of Cronbach’s α = .71 to Cronbach’s α = .76 [73].

##### Psychosocial adjustment: Child behavior

To assess the children’s emotional and behavioral problems during the last 12 months, the Strengths and Difficulties Questionnaire (SDQ) is utilized. On a 3-point Likert scale ranging from 0 “not true” to 2 “certainly true”, children are asked to rate problems relating to the subscales hyperactivity (with Cronbach’s α = .87) and conduct problems (α = .73) [74]. The questionnaire for the parents includes a proxy-report of their children’s emotional symptoms, conduct problems, hyperactivity, and peer problems with Cronbach’s α of .66, .60,.76, and α = .58, respectively [75].

#### Web-based extended measures for parents

##### Sociodemographic assessment

The parental socioeconomic status (SES) is measured using the MacArthur Scale [76] and Winkler-Index. The German version of the single-item MacArthur Scale [76] is applied to measure the parental subjective SES. Participants are asked to indicate their social rank on a 10-rung ladder, with the bottom representing people with the lowest education and least money and the top representing people with the highest education and most money. Three items of the Winkler-Index [77] are applied to measure the parent’s socioeconomic status by asking about the highest secondary education, professional degree, and employment status. The family’s SES is derived from an overall score, ranging from 3 to 21.

##### Psychosocial adjustment

In addition to the measures described above, the extended online survey for parents includes several other questionnaires to measure their psychosocial adjustment. As previously applied in the basic questionnaires, the WHO-5 measures the parents’ overall well-being. Further characteristics of the questionnaire are discussed in section “COVID-19-specific well-being in the everyday life”.

##### Psychosocial adjustment: Anxiety and depression

To assess parent’s anxiety and depression levels, the German version of the PHQ-4, the short version of the PHQ-9 and GAD-7 used for adolescents in the basic survey, is applied as screening instrument. Four Items are to be rated on a 4-point Likert scale ranging from 0 “not at all” to 3 “nearly every day”. An internal consistency of Cronbach’s α = .82 has been reported [78].

##### Psychosocial adjustment: Perceived stress

The frequency of distress experienced by the subject will be measured using four items with the German version of the Perceived-Stress Scale (PSS-4). On a 5-point Likert scale, parents are asked to indicate how often they felt stressed during the last year (e.g., “In the last 12 months, how often have you felt difficulties were piling up so high that you could not overcome them?“), with a higher score indicating higher chronic stress. The PSS-4 has an internal consistency of Cronbach’s α = .72 [79].

##### Psychosocial adjustment: Quality of life

This study applies 13 items of the German version of the Child Health Questionnaire Parent Form (CHQ) to assess the impact of the child’s health on the parental quality of life across several domains. On a 5-point scale, the emotional impact is assessed with 3 items (e.g., “How much emotional worry or concern did each of the following cause for you: your child’s physical health?“), the impact on the family with 6 items (e.g., “How often has your child’s health or behavior limited the types of activities you could do as a family?“), and family cohesion with one item (e.g., “In general, how would you rate your family’s ability to get along with one another?“). On a 4-point Likert scale, 3 items measure the impact of the child’s health on the leisure time (e.g., “Were you limited in the amount of time you had for your own needs because of your child’s emotional well-being?“). The German version of the CHQ reports an internal consistency of Cronbach’s α = .76 for parental emotional impact, and α = .81 for parental time impact. For the family impact subscale, an internal consistency of Cronbach’s α = .83 has been reported [80].

##### Coping and resources

The following instruments are used to assess the coping strategies and the resources and burdens of the parents:

##### Coping and resources: Parental disease management

To assess the parental coping style, the German short version of the coping orientation to problems experienced (Brief COPE) inventory [81] will be utilized. The questionnaire consists of 14 subscales with 2 items each, namely, active coping; planning; positive reframing; acceptance; humour; religion; emotional support; instrumental support; self-distraction; denial; venting; substance use; behavioural disengagement; and self-blame. Each subscale is represented by two items, measured on a four-point Likert scale ranging from “not at all “to “very much“. Higher scores indicate a greater use of the respective coping style. All scales reached Cronbach’s alpha α > .60.

##### Coping and resources: Self-efficacy

The German version of the General self-efficacy scale (in German: Allgemeine Selbstwirksamkeitsskala (ASKU)) [82] is applied to measure the parental expectations of being competent to deal with daily difficulties and obstacles. The inventory consists of 3 items (e.g., “I can rely on my own abilities in difficult situations“), answered on a 5-point scale ranging from 0 = “doesn’t apply at all” to 4 = “applies completely”. Findings indicate a good reliability of McDonalds’s omega = .81 to .86 [82].

##### Coping and resources: Locus of control

Measuring the internal and external sense of control, a four-item scale for the assessment of locus of control (In German: Internale-Externale-Kontrollüberzeugung-4 (IE-4)) [83] is administered. Parents are asked to rate the items (e.g., “If I work hard, I will succeed“) on a 5-point Likert scale ranging from 0 = “doesn’t apply at all” to 4 = “applies completely”. The IE-4 has a McDonalds Omega of .53 to .71 [84].

##### Coping and resources: Social support

The 3-item OSLO social support scale (OSS-3) is implemented to measure the level of social support that parents perceive. The questions assess how many close confidents parents have, the amount of concern they receive from others, and how accessible practical help is from their neighbours. The sum score of 3-8 is categorized as poor social support, from 9 to 11 as moderate social support, and 12-14 as strong social support. In a representative German population, the OSS-3 showed an internal consistency of Cronbach’s α = .64 [85].

##### Coping and resources: Child’s coping and resources

If their child cannot or does not want to participate themselves, parents are asked to rate the child’s coping and resources. Three items, taken from the COVID-19 Snapshot Monitoring questionnaire [86], are implemented. On a 7-point scale, reaching from 0 = “totally disagree “to 6 = “totally agree”, the proxy-report items assess whether the child suffers from not seeing their friends, whether it is happy to spend more time with the family, and whether it is overall coping well with the changes. Additionally, 6 self-constructed items based on the scales of the Resource Questionnaire for Children and Adolescents (in German: Fragebogen zu Ressourcen im Kindes- und Jugendalter (FRKJ 8–16)) [87] are presented on a 4-point scale to measure the child’s resources as a proxy-report. Ranging from “never true “to “aways true“, the parents are asked to rate their child’s empathy, self-efficacy, confidence, coherence, optimism, and locus of control.

##### Family relations and interactions

The following tools are used to consider the potential impact at family level.

##### Family relations and interactions: Parenting behaviour

To measure the risk of harmful parenting, 4 items originating from a study by Clemens et al. [88] is implemented. The items range on a 7-point Likert scale from 0 “does not apply at all “to 6 “applies very strongly“, asking the parents to indicate whether they are yelling more at the child, are more impatient, are more scared of slapping the child, or are more scared that their partner will slap the child since the beginning of the COVID-19 pandemic.

##### Family relations and interactions: Family-related quality of life

The survey includes 12 items assigned to two of three subscales of the family-related life questionnaire (FLQ) developed by Tröster [89]. Four items are ascribed to the subscale “relief from stress and self-fulfilment “and 8 items measure the subscale “social support within the family“. The 5-point scale ranges from 0 = “never/almost never “to 4 = “very frequently”, and the FLQ has an overall good internal consistency of Cronbach’s α = .93 to .94 [89].

##### Family relations and interactions: Parental burnout

The 5-item Brief Parental Burnout scale (BPBs) is applied to measure the risk of parental burnout. Parents are asked to rate the frequency of feelings related to burn-out (e.g., “I’m so tired out by my role as a parent that sleeping doesn’t seem like enough.“) on a 3-point response scale reaching from daily to more seldom/never. The BPBs reports an internal consistency of Cronbach’s α = .81 to .84 [90].

##### Control variables

The short form of the German Scale for Detecting Test Manipulation through Faking Good and Social Desirability Bias (in German: Skala zur Erfassung von Testverfälschung durch positive Selbstdarstellung und sozial erwünschte Antworttendenzen (SEA-K)) [91] is implemented to control for the social desirability bias. On a 4-point Likert scale, parents are asked to rate how much they agree with statements closely related to social desirability (e.g., “I would never talk behind my employer’s or colleague’s back“). The SEA-K has an internal consistency of Cronbach’s α = .59 [91].

#### Web-based extended measures for children

##### Psychosocial adjustment

To measure the child’s health related quality of life (HRQoL), 16 items of the KIDSCREEN-27 [92] are implemented. The participants are asked to indicate their HRQoL on 5 dimensions. The subscale “physical well-being “comprises 5 items (e.g., “Thinking about the last week, have you felt fit and well?“), 4 items are assigned to the dimension “social support and peers “(e.g., “Thinking about the last week, have you spent time with your friends?“). The internal consistency for each subscale of the KIDSCREEN-27 is Cronbach’s α > .70 [92]. Furthermore, the Kidscreen-10 index is applied to determine a global HRQoL score. The global score is derived from 10 items and has a good internal consistency of Cronbach’s α = .82 [92].

##### Coping and resources: Coping

Children’s coping strategies are assessed with the Coping with a disease questionnaire CODI [93]. Children indicate how frequently they use six coping styles on a 5-point Likert scale ranging from “never “to “always“. The items are assigned to the 6 subscales: acceptance, avoidance, cognitive-palliative, distance, emotional reaction, and wishful thinking, with internal consistencies ranging from α = .69 to α = .83 [93].

##### Coping and resources: Resources

To assess the children’s personal and social resources, the Questionnaire of Resources in Childhood and Youth (in German: Fragebogen zu Ressourcen im Kindes und Jugendalter (FRKJ-8-16)) is applied. The current study uses the subscales “sense of coherence“, “optimism“, “self-efficacy“, and “parental support“, comprising 6 items each (e.g., “I make sense of my life“). Responses are measured on 4-point Likert scales ranging from “never true “to “always true“. The internal consistency of the subscales varies between Cronbach’s α = 0.69 to α = 0.89 [87].

Based on self-constructed items from another study [59], 6 items are implemented to measure children’s corona-specific self-efficacy. Children are asked to rate the items (e.g., “I always know how to behave during the corona crisis”) on a 5-point Likert scale ranging from “not true at all” to “absolutely true”.

##### Coping and resources: Social integration

The survey includes 4 items based on self-constructed items from another study [59] to measure the amount of time children spend with their peers during and before the pandemic. On a 5-point Likert scale ranging from “very rarely “to “very often“, children are asked to answer how often they are meeting their friends, how often they met before the pandemic, how often they communicate digitally, and how often they used to communicate before the pandemic.

##### Family relations and interactions: Quality of child-parent relationship

The German version of the Parental-Representation-Screening-Questionnaire (PRSQ), in German “Elternbildfragebogen für Kinder und Jugendliche “(EBF-KJ), is administered to assess how the children rate the relationship with their parents. This survey uses two modified scales of the EBF-KJ, namely “autonomy “and “overprotection“, asking children to answer 8 items on 5-point Likert scales. The internal consistency of the subscales ranges from Cronbach’s α = .72 to .85 for patients [94].

##### Family relations and interactions: COVID-19-specific social support

To assess the perceived support during the COVID-19 pandemic, 8 self-constructed items are applied. On a scale ranging from 0 “completely disagree “to 4 “completely agree“, children are asked how much they have talked about the coronavirus with their parents (e.g., “My parents have explained to me what COVID-19 is“) and their friends (e.g., “I have talked to my friends about COVID-19″).

### Sample size

With respect to the baseline survey, we will implement a convenience sampling procedure: That means that all facilities taking part in one of the three patient registries have been informed about the ongoing KICK-COVID study via newsletters and regular meetings of the collaborators. We aim to include (and re-assess) as many participants as possible in order to get sufficient data sets to make generalizable statements. Therefore, we intend to recruit at least 1000 parents of either children or adolescents per CC. In addition, we assume that around 500 adolescents (> 12 yrs) per CC will fill in the baseline survey. For the web-based psychosocial assessment, the primary goal is to get a deeper understanding of the assumed psychosocial processes that can explain the level of mental and physical strain experienced. Therefore, we aim to include a subsample of participants of the baseline survey. To apply structural equation modeling (SEM) required sample sizes are dependent on various data characteristics such as data distribution, the number of missing data, or parameters that have to be computed. According to Kline [95], the sample size should fall above the number of 200 although sample sizes with at least 100 participants might be sufficient. Since drop-out rates vary between 0 and 54% [96] we aim to include between 300 and 400 parents and about 100 adolescents.

### Statistical analyses

In accordance with common guidelines, we will only include online survey data with a high data quality indication but a “realistic” completion time of the questionnaire (a relative speed indicator higher than 2 and a total completion time of at least 5 minutes [97]). With respect to missing data and drop-outs, multiple imputations via fully conditional specification implemented will be performed [98]. We will apply multivariate ANOVAs and (logistic, hierarchal) regression analyses to analyze disease-specific differences with respect to health care use, risk perception and mental health. Sociodemographic variables such as age and sex of the child, disease severity, and risk perception will be included as predictors, as well as the date the questionnaire is completed as indicator of the stringency of current containment measures. To analyze the interplay between physical and mental health and the impact of corona-related stressors, SEM will be applied. All analyses will be conducted using SPSS, SAS; R or Mplus.

## Discussion

The COVID-19 pandemic has led to far-reaching changes in everyday life for everyone worldwide. Families with underage young people in particular are and were affected in many ways: be it the closure of schools and daycare centers with the resulting need for homeschooling or home care, far-reaching contact restrictions and lockdowns, or the changed working situation of parents with home offices or part-time work, to name just a few measures that aimed to curb the further spread of the contagious virus. These measures have not spared the health system with the (partial) closure of outpatient clinics, limited contact times, or the establishment of video consultation hours. It is precisely these restrictions that affect those who are most in need of regular medical and psychosocial care: people with CCs.

Since the outbreak of the pandemic, numerous studies have examined the consequences for society. There is now meta-analytical evidence on the psychological consequences for children and adolescents [33, 46, 99] and their parents [100]. The psychosocial effects of the COVID-19 pandemic on children and their parents have also been and are being extensively examined for the German-speaking area, e.g., with the COPSY study [101] or the LIFE Child study [102]. In contrast, KICK-COVID focuses explicitly on the group of children with a chronic illness and their parents. With the help of three patient registers, not only medical outcomes but also psychosocial outcomes and their interplay can be examined comprehensively.

### Strengths and limitations

To the best of our knowledge, no other national study focuses on the group of children and adolescents with a chronic illness during the COVID-19 pandemic.

The KICK-COVID study and its expected results must be viewed in light of its strengths and weaknesses. A major strength of our approach is the large expected sample size. The short economic questionnaire aims to reach as many of those affected as possible. The large sample size makes it possible to make statements that are as representative as possible, not only in relation to the respective underlying physical disease, but also across the disease groups in order to identify generic and condition-specific patterns. In addition, the extended psychosocial web survey will enrich our database by providing more detailed information on the psychosocial situation and coping strategies of the families. Of note, a follow-up over 1 year will allow us to examine short and longer-term effects and different trajectories over time.

With respect to the limitations of the study, it should be mentioned that a self-selection of interested clinicians and patients cannot be ruled out. Thus, clinicians might not approach certain patients, either because they may perceive these persons as too distressed or because they believe that the afflicted patients do not have any problems. Especially, a bias regarding the inclusion of the high-risk group in the obesity sample can be expected for the basic survey and the extended survey in particular. Due to organizational reasons, we are not able to implement the questionnaires as obligatory, and we could not offer monetary compensation for the clinics and patients to mitigate this effect. However, we are aiming for a large sample that may be able to compensate for this effect. Furthermore, the data already available from the registers allow us to characterize our sample in relation to the total population. Of course, self-selection could be even more pronounced when parents are invited to fill in the questionnaire. Due to ethical reasons and German data protection law regulations, we have to ask the parents to forward the children’s questionnaire with their consent to their children.

Taken together, the results of the proposed study will provide empirical data on how to support families with a child suffering from a CC and contribute to a more successful and sustainable health system. The identification of determining factors for positive adjustment will offer new opportunities for interventions. The prospective design will not only allow the detection of acute and delayed long-term effects, but also the analysis of mediating and moderator influences.

## Data Availability

Fully anonymized data will be available from the corresponding author on reasonable request and with the permission of the collaboration partners.

## Abbreviations

APV: German Obesity Prospective Follow-up Registry
ASKU: General self-efficacy scale (in German: Allgemeine Selbstwirksamkeitsskala)
BPBs: Brief Parental Burnout scale
Brief COPE: Short version of the coping orientation to problems experienced inventory
CC: Chronic physical health condition
CHQ: Child Health Questionnaire
CODI: Coping with a disease questionnaire
DPV: German Diabetes Prospective Follow-up Registry obesity
EBF-KJ: German Parental-Representation-Screening-Questionnaire (in German: Elternbildfragebogen für Kinder und Jugendliche)
FLQ: Family-related life questionnaire
FRKJ-8-16: Questionnaire of Resources in Childhood and Youth (in German: Fragebogen zu Ressourcen im Kindes und Jugendalter)
GAD-7: General Anxiety Disorder-Scale
HRQoL: Health related quality of life
IE-4: Assessment of locus of control (In German: Internale-Externale-Kontrollüberzeugung-4)
NPRD: National Pediatric Rheumatology Database
OSS-3: OSLO social support scale
PHQ-9: 9-item Patient Health Questionnaire
PRSQ: Parental Representation-Screening-Questionnaire (for the German version see also EBF-KJ)
PSS-4: Perceived-Stress Scale
SDQ: Strengths and Difficulties Questionnaire
SEA-K: Scale for Detecting Test Manipulation through Faking Good and Social Desirability Bias (in German: Skala zur Erfassung von Testverfälschung durch positive Selbstdarstellung und sozial erwünschte Antworttendenzen)
SEM: Structural equation modeling
SES: Socioeconomic status
WHO-5: World Health Organization Five Well-Being Index
